# Surgical Aid Society

**Published:** 1924-01

**Authors:** 


### SURGICAL AID SOCIETY.

THE sixty-first annual general meeting of the Royal Surgical Aid Society was held by favour of the Lord Mayor at the Mansion House on Wednesday, December 12. The society was established in 1862 to supply surgical appliances to the poor. The record of the sixty-one years is one which may be reviewed with pride and satisfaction, and that of the past twelve months is most encouraging both in regard to work achieved and the financial support accorded. During its existence the society has supplied 1,149,229 surgical appliances to 748,211 patients.

### A BENEFICENT WORK.

There are eighteen local branches, each having its own committee, secretary and surgeon, and no limitations as to creed, nationality, character or complaint are imposed. Those needing assistance have simply to procure a doctor's certificate of what is required and then obtain from subscribers a sufficient number of letters to cover the cost of the appliance. Special grants of letters are made by the committee when the patient cannot obtain the required number, and thus all deserving applicants receive assistance without delay. The year's income shows an increase of £2,519 on that of the previous year, and the deficit has been greatly reduced, but increased financial support is much needed. The Committee of Management, which meets once a month, is indefatigable in its efforts to render all the help possible, and it is to be hoped that so gratifying a report as that presented at the meeting is a harbinger of coming growth and prosperity and that the society may go on from success to success for many years to come.

